# Association between the fibrosis-4 and the risk of left ventricular aneurysm formation in acute ST-segment elevation myocardial infarction

**DOI:** 10.3389/fcvm.2025.1477206

**Published:** 2025-06-13

**Authors:** Kai Zhang, Lihong Yang, Yonghui Zhao

**Affiliations:** ^1^ People's Hospital of Zhengzhou University, Fuwai Central China Cardiovascular Hospital, Henan Provincial People's Hospital, Zhengzhou, Henan, China; ^2^ Henan Provincial People's Hospital, Zhengzhou, Henan, China

**Keywords:** acute ST-segment elevation myocardial infarction, primary percutaneous coronary intervention, left ventricular aneurysm, fibrosis-4 index, risk

## Abstract

**Background:**

Left ventricular aneurysm (LVA) often occurs as a complication following an acute myocardial infarction. This research focused on assessing the ability of the fibrosis-4 (FIB-4) index to predict LVA formation in individuals with acute ST-segment elevation myocardial infarction (STEMI) who underwent primary percutaneous coronary intervention (PCI).

**Methods:**

We included 1,384 consecutive patients diagnosed with STEMI and compared their clinical and laboratory data between the LVA group and the non-LVA group. To determine the independent risk factors for LVA formation, multivariable logistic regression analysis was employed. Restricted cubic spline (RCS) analysis was conducted to evaluate the nonlinear relationship between FIB-4 index and LVA formation. The ROC curve was used to determine the predictive capability of the FIB-4 index and composite variable for LVA formation.

**Results:**

LVA occurred in 12.7% of the population. An elevated FIB-4 index correlated with a higher occurrence of LVA (19% vs. 9.3%, *P* < 0.001). In the LVA group, the FIB-4 index was higher than in the non-LVA group [1.8 (1.1–4.6) vs. 3.5 (1.4–8.1), *P* < 0.001]. Analysis using multivariable logistic regression showed that the FIB-4 index independently correlated with LVA risk (OR =  1.73, *P* = 0.004). The analysis using RCS uncovered a nonlinear correlation between a higher FIB-4 index and a heightened risk of LVA (Nonlinear *P* = 0.009). Additionally, the area under the ROC curve for the FIB-4 index in predicting LVA was 0.617. The composite variable comprising the FIB-4 index, left ventricular ejection fraction (LVEF), estimated glomerular filtration rate (eGFR), and left anterior descending artery (LAD) as culprit vessel significantly improved the predictive power (C statistic = 0.722).

**Conclusion:**

An increased FIB-4 index was positively associated with LVA formation in patients with acute STEMI who underwent primary PCI.

## Introduction

Left ventricular aneurysm (LVA) is characterized by an abnormal protrusion of the left ventricular wall, which occurs during both systole and diastole as a result of localized myocardial necrosis and fibrosis following myocardial infarction (1). This is a typical complication associated with acute myocardial infarction (AMI), with an incidence ranging from 5% to 15% (2). Patients with LVA formation are more likely to experience arrhythmias (3), thromboembolic events (4), cardiac rupture, and potentially fatal outcomes (5). A previous study has demonstrated that the risk of cardiovascular death was doubled in AMI patients with LVA compared to those without (6). Therefore, predicting LVA formation early is vital for prompt diagnosis and treatment, with the goal of lowering the mortality rate in AMI patients.

The fibrosis-4 (FIB-4) index, derived from age, platelet count, and the levels of aspartate aminotransferase (AST) and alanine aminotransferase (ALT), serves as an easy and non-invasive marker for liver fibrosis (7). A substantial body of clinical research has demonstrated a robust relationship between the FIB-4 index and cardiovascular diseases (CVDs). A retrospective cohort study of 81,108 participants with nonalcoholic fatty liver disease identified the FIB-4 index as a key independent predictor of major adverse cardiovascular events (8). A study by Takae et al. indicated that the FIB-4 index is an effective predictor of total cardiovascular events in patients with preserved left ventricular ejection fraction (9). A higher FIB-4 index has been linked to a greater risk of ischemic heart disease in a general population followed up for 10 years (7). Additionally, individuals with a higher FIB-4 index exhibited an increased risk of all-cause mortality among patients with AMI (10). To date, no studies have focused on the FIB-4 index's ability to predict LVA formation in patients suffering from AMI. The objective of this study is to investigate the relationship between the FIB-4 index and the risk of LVA formation in patients with acute ST-segment elevation myocardial infarction (STEMI).

## Methods

### Study subjects

**Figure 1:**
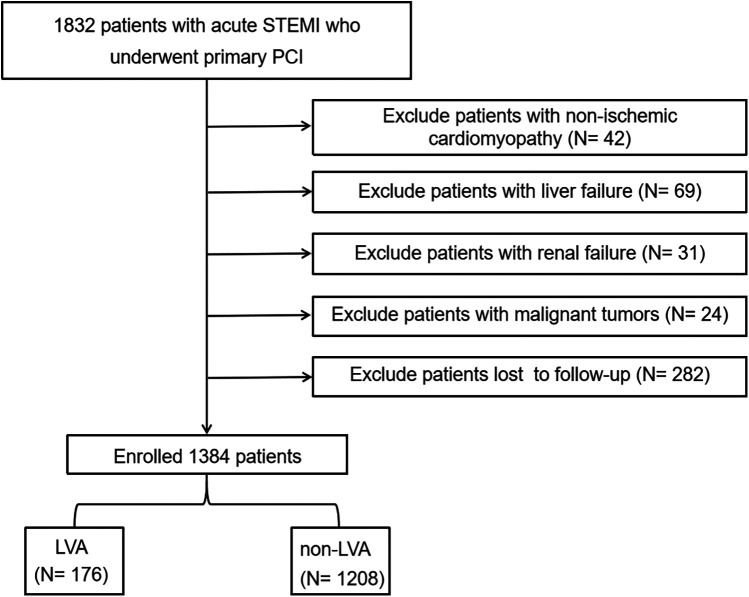
Flow diagram of the study participants. STEMI, acute ST-segment elevation myocardial infarction; PCI, percutaneous coronary intervention; LVA, left ventricular aneurysm.

This investigation complied with the Declaration of Helsinki principles and was approved by the Central China Fuwai Hospital's Review Board, with all patients giving informed consent before taking part. For this prospective study, we recruited 1,832 consecutive acute STEMI patients who underwent primary PCI at Central China Fuwai Hospital from 2018 to 2024. Acute STEMI was determined by the criteria outlined in the fourth universal definition of myocardial infarction (11), which encompasses the following criteria: typical chest pain persisting for over 30 minutes, with new ST-segment elevation at the J point in at least two contiguous leads of >2 mm (0.2 mV) in men or >1.5 mm (0.15 mV) in women on admission electrocardiogram, and an increase in cardiac enzyme levels above the 99th percentile cut-off point for cardiac troponin I (cTnI). Individuals were excluded if they had congenital heart disease, non-ischemic cardiomyopathy (such as hypertrophic and dilated cardiomyopathy), failure of the kidneys or liver, active infection, malignant tumors, or a life expectancy of less than one year, or if they had received thrombolytic therapy prior to admission, or loss to follow-up. Finally, the analysis involved a total of 1,384 patients (Figure 1).

### Data collection and definitions

Physicians who were unaware of the study's objectives gathered patient demographic and clinical data from the electronic medical records, such as age, gender, hypertension, diabetes, and medication prescribed upon discharge. The diagnosis of hypertension and diabetes have been previously described (1). Between 7:00 and 9:00 a.m., blood samples were taken from the elbow vein of every patient admitted to the emergency room. The FIB-4 index was calculated using the following formula: FIB-4 index = age (years) × AST (U/L)/[ALT (U/L)^1/2^ × platelet count (10^9^/L)] (12).

### Echocardiography and PCI procedure

For all patients, two-dimensional transthoracic echocardiography (TTE) was carried out within three days of admission and at the end of the first and sixth months during follow-up. Diagnosis of LVA was established using the criteria from the Coronary Artery Surgery Study (CASS) (13). Criteria for LVA diagnosis were: (I) bulging of the left ventricular wall during diastole and systole with either akinesia or dyskinesia; (II) a distinct boundary of the infarcted segment; and (III) no trabeculation present in the affected segment. The procedural details of percutaneous coronary intervention (PCI) have been documented in prior studies (1).

### Statistical analysis

Statistical analysis was performed utilizing SPSS version 13.0 (SPSS, Inc, Chicago, Illinois) for the Windows operating system (Microsoft Corp, Redmond,Washington). In terms of data presentation, categorical variables were summarized as counts accompanied by their respective percentages. For continuous variables, the presentation varied based on the distribution of the data; these variables were represented either as the mean ± standard deviation or the median with interquartile range, depending on whether the data adhered to a normal distribution. Through ROC curve analysis, the optimal FIB-4 index cut-off for dividing patients into two groups was set at 3.25. Group differences were evaluated using the chi-squared test for categorical data, the independent-sample *t*-test for normally distributed continuous data, and the Mann–Whitney *U*-test for continuous data with a skewed distribution. Univariate logistic regression analysis was performed to explore the relationship between different variables and the risk of LVA formation. The multivariate logistic regression analysis incorporated variables that demonstrated a significance level of *P* < 0.05 during the univariate analysis. All comparisons conducted within this analysis were two-sided. Statistical significance was firmly established at a threshold of *p* < 0.05.

## Results

### Patient characteristics

Our research comprised 1,384 individuals diagnosed with acute STEMI who received primary PC. The average age of the participants in this study was 60.4 ± 12.6, with males making up 75.9% of the cohort. Throughout the follow-up period involving TTE, we observed 176 instances (12.7%) of LVA.

Participants were categorized into two groups: the LVA group (*N* = 176) and the no-LVA group (*N* = 1,208). As presented in Table 1, individuals in the LVA group were generally older and exhibited elevated levels of left ventricular end-diastolic diameter (LVEDD), white blood cell count, platelet count, as well as higher values for AST, glycated hemoglobin (HbA1c), lactate dehydrogenase (LDH), N-terminal pro-B-type natriuretic peptide (NT-proBNP), and the FIB-4 index. Additionally, they demonstrated a greater prevalence of hypertension, diabetes, and LAD as culprit vessel (*P* < 0.05). In contrast, the LVA group displayed reduced left ventricular ejection fraction (LVEF) and estimated glomerular filtration rate (eGFR) when compared to those without LVA (*P* < 0.05).

**Table 1:** Baseline characteristics of patients stratified by the presence of left ventricular aneurysm.

Variables	Whole cohort (*N* = 1,384)	Non-LVA patients (*N* = 1,208)	LVA patients (*N* = 176)	*P*-value
Demographics				
Age, years	60.4 ± 12.6	60.0 ± 12.6	63.4 ± 12.3	<0.001
Male, *n* (%)	1,050 (75.9)	921 (76.2)	129 (73.3)	0.39
Hypertension, *n* (%)	673 (48.6)	567 (46.9)	106 (60.2)	0.001
Diabetes, *n* (%)	403 (29.1)	332 (27.5)	71 (40.3)	<0.001
LVEF, %	48.1 ± 11.7	49.1 ± 11.7	41.6 ± 9.8	<0.001
LVEDD, mm	49.8 ± 6.5	49.5 ± 6.3	51.8 ± 7.2	<0.001
BMI, kg/m^2^	25.4 ± 3.3	25.4 ± 3.3	25.2 ± 3.4	0.532
Laboratory parameters				
White blood cell count, × 10^9^/L	8.5 (6.5-11.3)	8.4 (6.5-11.0)	9.2 (6.7-12.8)	0.02
Platelet count, × 109/L	235.2 ± 73.7	233.1 ± 70.6	249.6 ± 90.9	0.005
ALT, U/L	27.7 (11.4-76.4)	28.5 (11.6-77.4)	24.2 (10.6-66.5)	0.262
AST, U/L	42.1 (22.6-114.3)	40.25 (22.00, 99.00)	66.20 (29.95, 177.65)	<0.001
ALB, g/L	41.3 ± 8.4	41.4 ± 8.2	40.9 ± 9.3	0.54
HbA1c, %	5.8 (5.5-7.0)	5.8 (5.5-7.0)	5.9 (5.6-7.1)	0.019
TC, mmol/L	4.8 ± 1.2	4.8 ± 1.3	4.8 ± 1.0	0.647
TG, mmol/L	1.4 (1.0-1.9)	1.4 (1.0-1.9)	1.4 (1.1-1.9)	0.848
HDL, mmol/L	1.0 ± 2.2	1.1 ± 2.3	0.9 ± 0.2	0.488
LDL, mmol/L	3.2 ± 0.9	3.2 ± 0.9	3.2 ± 0.9	0.764
LDH, U/L	325 (212-658)	316 (210-623)	393 (236-822)	0.017
eGFR, ml/min/1.73m^2^	82.6 ± 21.6	83.9 ± 20.9	73.4 ± 23.8	<0.001
NTproBNP, pg/ml	1,435 (537-3,321)	1,282 (486-2,921)	2,580 (1,387-5,000)	<0.001
FIB-4	1.9 (1.1-5.0)	1.8 (1.1-4.6)	3.5 (1.4-8.1)	<0.001
Medication at hospital discharge				
Statin, *n* (%)	1,317 (95.2)	1,154 (95.5)	163 (92.6)	0.092
β-blockers, *n* (%)	1,295 (93.6)	1,133 (93.8)	162 (92.0)	0.378
ACE inhibitors or ARB, *n* (%)	943 (68.1)	824 (68.2)	119 (67.6)	0.874
Coronary artery injure				
LAD as Culprit vessel, (%)	1,114 (80.5)	959 (79.4)	155 (88.1)	0.007
Multiple vessel disease, (%)	866 (62.6)	759 (62.8)	107 (60.8)	0.602

LVEF, left ventricular ejection fraction; LVEDD, left ventricular end-diastolic diameter; BMI, body mass index; ALT, alanine aminotransferase; AST, aspartate aminotransferase; ALB, albumin; HbA1c, glycated hemoglobin; TC, total cholesterol; TG, triglyceride; HDL, high-density lipoprotein; LDL, low-density lipoprotein; LDH, Lactate dehydrogenase; eGFR, estimated glomerular filtration rate; NT-proBNP, N-terminal pro-B-type natriuretic peptide; ACE, Angiotensin converting enzym; ARB, angiotensin receptor blocker; LAD, Left anterior descending artery.

Utilizing the criterion of the Maximum Youden Index, we determined the optimal cut-off value for the FIB-4 index to be 3.25, which allows for the stratification of patients into two categories: FIB-4 < 3.25 and FIB-4 ≥ 3.25. Table 2 illustrates that individuals in the FIB-4 ≥ 3.25 group were generally older and exhibited elevated levels of white blood cell count, ALT, AST, albumin (ALB), HbA1c, LDH, and NT-proBNP, alongside a greater prevalence of diabetes and left ventricular aneurysm (LVA) formation. Conversely, patients with FIB-4 ≥ 3.25 demonstrated reduced levels of LVEF, platelet count, and eGFR, and were also less inclined to be prescribed angiotensin-converting enzyme (ACE) inhibitors or angiotensin II receptor blockers (ARBs) when compared to those with FIB-4 < 3.25.

**Table 2:** Baseline characteristics of patients stratified by FIB-4 level.

Variables	FIB-4 < 3.25 (*N* = 895)	FIB-4 ≥ 3.25 (*N* = 489)	*P*-value
Demographics			
Age, years	59.1 ± 12.7	62.8 ± 12.1	<0.001
Male, *n* (%)	677 (75.6)	373 (76.3)	0.792
Hypertension, *n* (%)	430 (48.0)	243 (49.7)	0.558
Diabetes, *n* (%)	232 (25.9)	171 (35.0)	<0.001
LVEF, %	50.5 ± 11.2	43.8 ± 11.5	<0.001
LVEDD, mm	49.7 ± 6.3	49.9 ± 6.7	0.698
BMI, kg/m^2^	25.5 ± 3.4	25.2 ± 3.1	0.097
Laboratory parameters			
White blood cell count, × 10^9^/L	7.6 (6.1-9.6)	10.8 (8.3-14.1)	<0.001
Platelet count, × 10^9^/L	238.9 ± 67.6	228.3 ± 83.4	<0.001
ALT, U/L	29 (18–50)	46.2 (31–83.7)	<0.001
AST, U/L	25.0 (16.0–42.4)	144.4 (89.8–219.3)	<0.001
ALB, g/L	42.6 ± 8.4	38.9 ± 7.9	<0.001
HbA1c, %	5.8 (5.4–6.7)	6.0 (5.5–7.5)	<0.001
TC, mmol/L	4.8 ± 1.3	4.8 ± 1.0	0.935
TG, mmol/L	1.4 (1.0–1.9)	1.4 (1.0–1.8)	0.177
HDL, mmol/L	1.0 ± 0.3	1.0 ± 0.3	0.135
LDL, mmol/L	3.2 ± 0.9	3.2 ± 0.8	0.781
LDH, U/L	256 (188–409)	610 (340–1,113)	<0.001
eGFR, ml/min/1.73m^2^	85.4 ± 19.7	77.3 ± 23.8	<0.001
NT-proBNP, pg/ml	1,038 (384.2–2,430)	2,460 (1,240–5,029.5)	<0.001
FIB-4	1.3 (0.9–1.9)	7.0 (4.7–11.4)	<0.001
Medication at hospital discharge			
Statin, *n* (%)	851 (95.1)	466 (95.3)	0.86
β-blockers, *n* (%)	839 (93.7)	456 (93.3)	0.722
ACE inhibitors or ARB, *n* (%)	635 (70.9)	308 (63.0)	0.002
Coronary artery injure			
LAD as Culprit vessel, (%)	708 (79.1)	406 (83.0)	0.079
Multiple vessel disease, (%)	547 (61.1)	319 (65.2)	0.13
LVA, *n* (%)	83 (9.3)	93 (19.0)	<0.001

LVEF, left ventricular ejection fraction; LVEDD, left ventricular end-diastolic diameter; BMI, Body Mass Index; ALT, alanine aminotransferase; AST, aspartate aminotransferase; ALB, albumin; HbA1c, glycated hemoglobin; TC, total cholesterol; TG, triglyceride; HDL, high-density lipoprotein; LDL, low-density lipoprotein; LDH, Lactate dehydrogenase; eGFR, estimated glomerular filtration rate; NT-proBNP, N-terminal pro-B-type natriuretic peptide; ACE, Angiotensin converting enzym; ARB, angiotensin receptor blocker; LAD, left anterior descending artery; LVA, left ventricular aneurysm.

### FIB-4 index and the incidence of LVA

**Figure 2:**
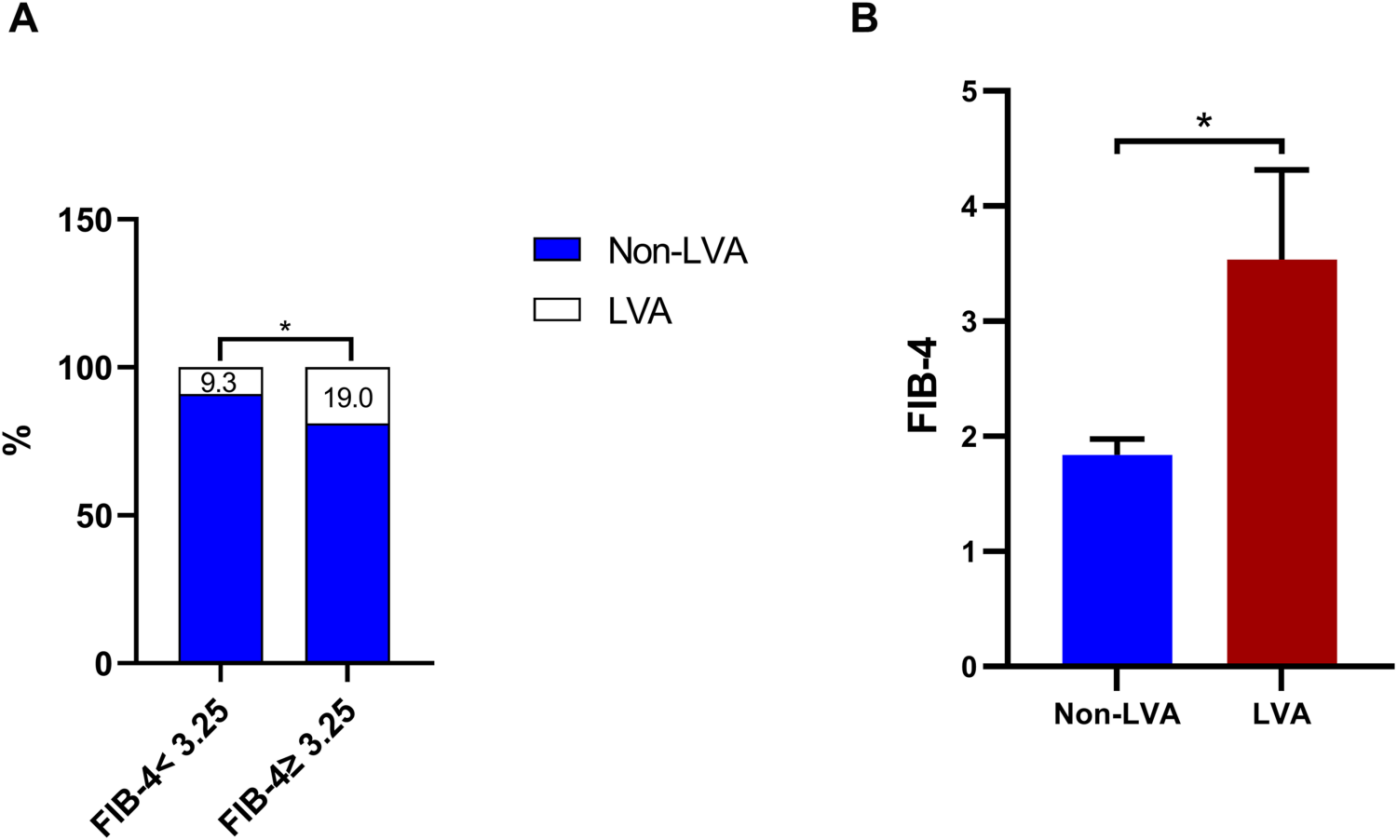
The effect of the FIB-4 index on the prevalence of LVA **(A)** and comparison of the FIB-4 index level between the LVA and non-LVA groups **(B)** FIB-4, fibrosis-4; LVA, left ventricular aneurysm; * *P* < 0.05.

The data illustrated in Figures 2A,B indicate that the formation of LVA occurred more frequently as the FIB-4 index increased (9.3% vs. 19%, *P* < 0.001). Additionally, the group with LVA showed a considerably elevated FIB-4 index in comparison to the group without LVA [3.5 (1.4–8.1) vs. 1.8 (1.1–4.6), *P* < 0.001].

**Figure 3:**
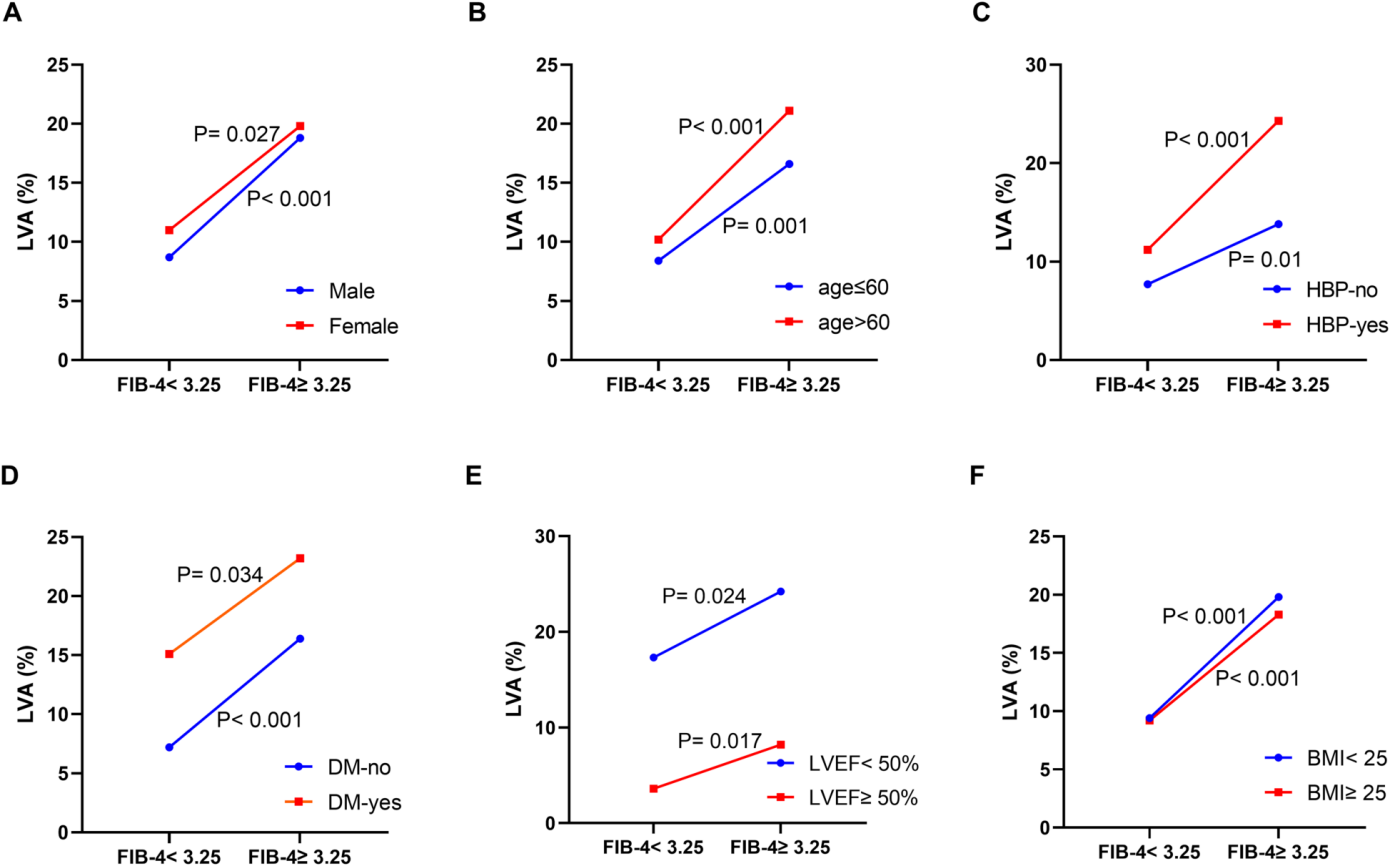
The impact of the FIB-4 index on the prevalence of LVA across subgroups of gender **(A)**, age **(B)**, HBP status **(C)**, DM status **(D)**, LVEF **(E),** and BMI **(F)** FIB-4, fibrosis-4; LVA, left ventricular aneurysm; HBP, hypertension; DM, diabetes mellitus; LVEF, left ventricular ejection fraction; BMI, body mass Index.

Additionally, we examined the relationship between the FIB-4 index and the prevalence of LVA across various subgroups. The data presented in Figures 3A–F demonstrate a correlation between increased FIB-4 index and higher incidence of LVA in both males (8.7% vs. 18.8%, *P* < 0.001) and females (11% vs. 19.8%, *P* = 0.027), individuals aged ≤ 60 (8.4% vs. 16.6%, *P* = 0.001) and those >60 (10.2% vs. 21.1%, *P* < 0.001), non-hypertensive (7.7% vs. 13.8%, *P* = 0.01) and hypertensive (11.2% vs. 24.3%, *P* < 0.001), non-diabetics (7.2% vs. 16.4%, *P* < 0.001) and diabetics (15.1% vs. 23.2%, *P* = 0.034), individuals with LVEF < 50% (17.3% vs. 24.2%, *P* = 0.024) and LVEF ≥ 50% (3.6% vs. 8.2%, *P* = 0.017), those BMI < 25 (9.4% vs. 19.8%, *P* < 0.001) and BMI ≥ 25 (9.2% vs. 18.3%, *P* < 0.001).

### Logistic regression analyses

We performed logistic regression analysis to evaluate the potential risk factors linked to the development of LVA. In the univariate logistic regression, a noteworthy association was identified between LVA and various parameters such as age, hypertension, diabetes, LVEF, LVEDD, white blood cell count, platelet count, HDL, eGFR, NT-proBNP, LAD as the culprit vessel, and the FIB-4 index (Table 3). Following this, a multivariate logistic regression analysis was performed, incorporating these 12 variables, which indicated that only LVEF (OR = 0.961, 95% CI = 0.944–0.978, *P* < 0.001), eGFR (OR = 0.989, 95% CI = 0.981–0.998, *P* = 0.012), LAD as the culprit vessel (OR =  1.837, 95% CI = 1.118–3.019, *P* = 0.016), and FIB-4 index (OR = 1.776, 95% CI = 1.225–2.575, *P* = 0.002) showed a significant correlation with the risk of developing LVA (Table 3).

**Table 3:** Logistic regression analysis.

Variables	Univariate logistic regression analysis			Multivariate logistic regression analysis		
	OR	95% CI	*P*-value	OR	95% CI	*P*-value
Age	1.023	1.009–1.036	<0.001			
Hypertension	1.712	1.24–2.363	0.001			
Diabetes	1.784	1.287–2.473	<0.001			
LVEF	0.948	0.935–0.961	<0.001	0.961	0.944–0.978	<0.001
LVEDD	1.051	1.027–1.075	<0.001			
White blood cell count	1.054	1.018–1.092	0.003			
Platelet count	1.003	1.001–1.005	0.006			
HDL	0.45	0.236–0.859	0.015			
eGFR	0.979	0.973–0.986	<0.001	0.989	0.981–0.998	0.012
NTproBNP	1	1.000–1.000	0.003			
LAD as Culprit vessel	1.916	1.19–3.087	0.007	1.837	1.118–3.019	0.016
FIB-4 (<3.25/≥3.25)	2.3	1.67–3.16	<0.001	1.776	1.225–2.575	0.002

LVEF, left ventricular ejection fraction; LVEDD, left ventricular end-diastolic diameter; HDL, high-density lipoprotein; eGFR, estimated glomerular filtration rate; NT-proBNP, N-terminal pro-B-type natriuretic peptide; LAD, Left anterior descending artery; FIB-4, fibrosis-4; OR, odds ratio.

**Figure 4:**
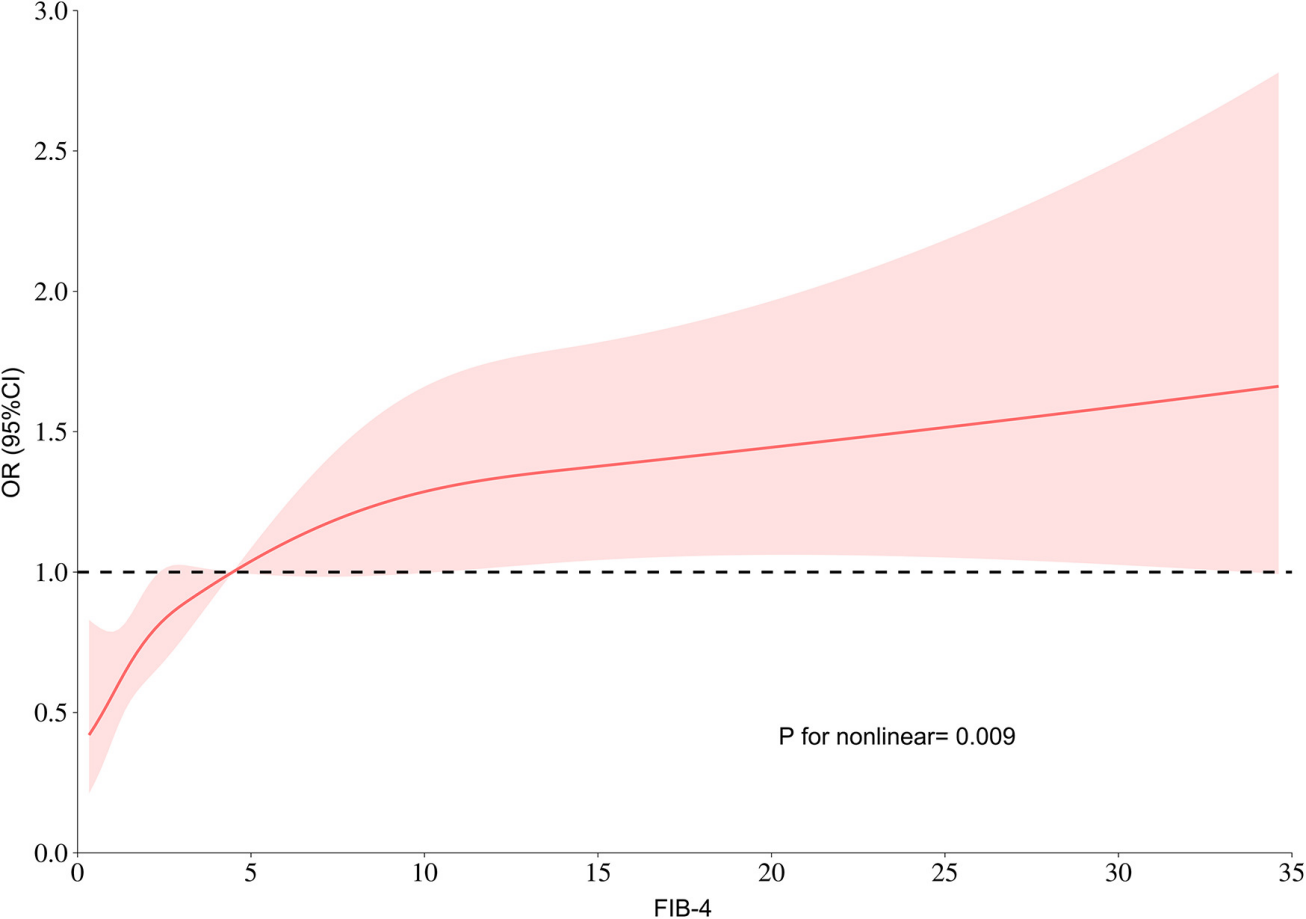
RCS model showing the links between the FIB-4 index and the risk of LVA. FIB-4, fibrosis-4; LVA, left ventricular aneurysm; OR, odds ratio.

Additionally, RCS was performed to model and visually represent the connection between the FIB-4 index and the risk of LVA. As illustrated in Figure 4, the findings reveal a positive nonlinear correlation between the FIB-4 index and the formation of LVA. This correlation persists even after adjusting for other potential risk factors such as age, hypertension, and diabetes (Nonlinear *P* = 0.009).

### Subgroup analysis

**Figure 5:**
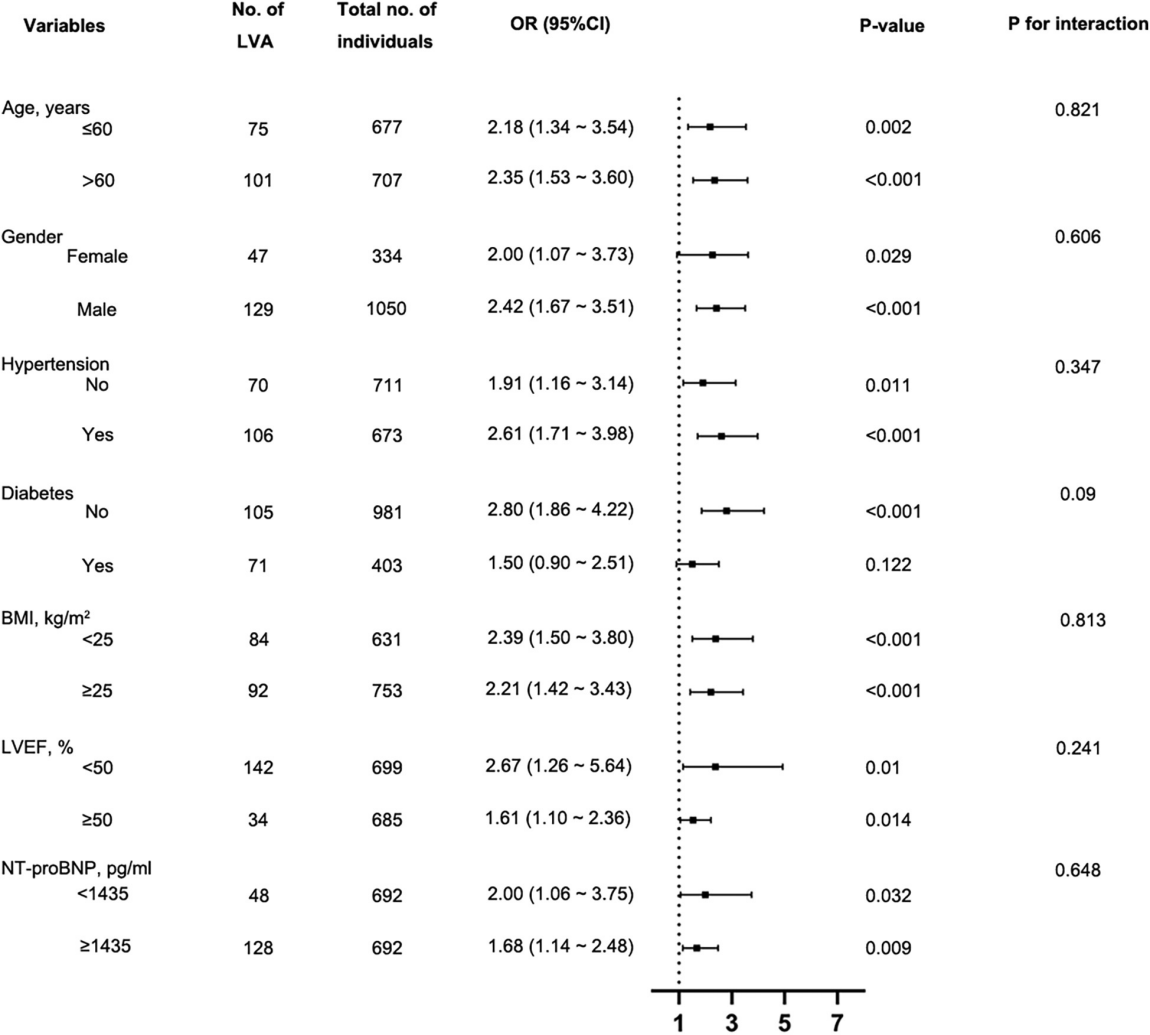
Subgroup analysis using forest plots to assess the connection between the FIB-4 index and the likelihood of LVA formation. FIB-4, fibrosis-4; LVA, left ventricular aneurysm; LVEF, left ventricular ejection fraction; BMI, Body Mass Index; NT-proBNP, N-terminal pro-B-type natriuretic peptide; OR, Odds Ratio.

We evaluated the independent relationship between the FIB-4 index and LVA formation in different clinically relevant subgroups (Figure 5). The FIB-4 index was significantly associated with the risk of LVA formation in the subgroups of males and females, individuals with age ≤ 60 and >65 years, with and without hypertension, without diabetes, individuals with BMI < 25 kg/m^2^ and ≥25 kg/m^2^, with LVEF < 50% and LVEF ≥ 50%, as well as individuals with NT-proBNP < 1,435 pg/ml and ≥1,435 pg/ml. Besides, no notable interactions were identified between the FIB-4 index and these subgroups with regard to the risk of LVA (P for interaction > 0.05).

### Association between FIB-4 index and echocardiography parameters of LVA

**Figure 6:**
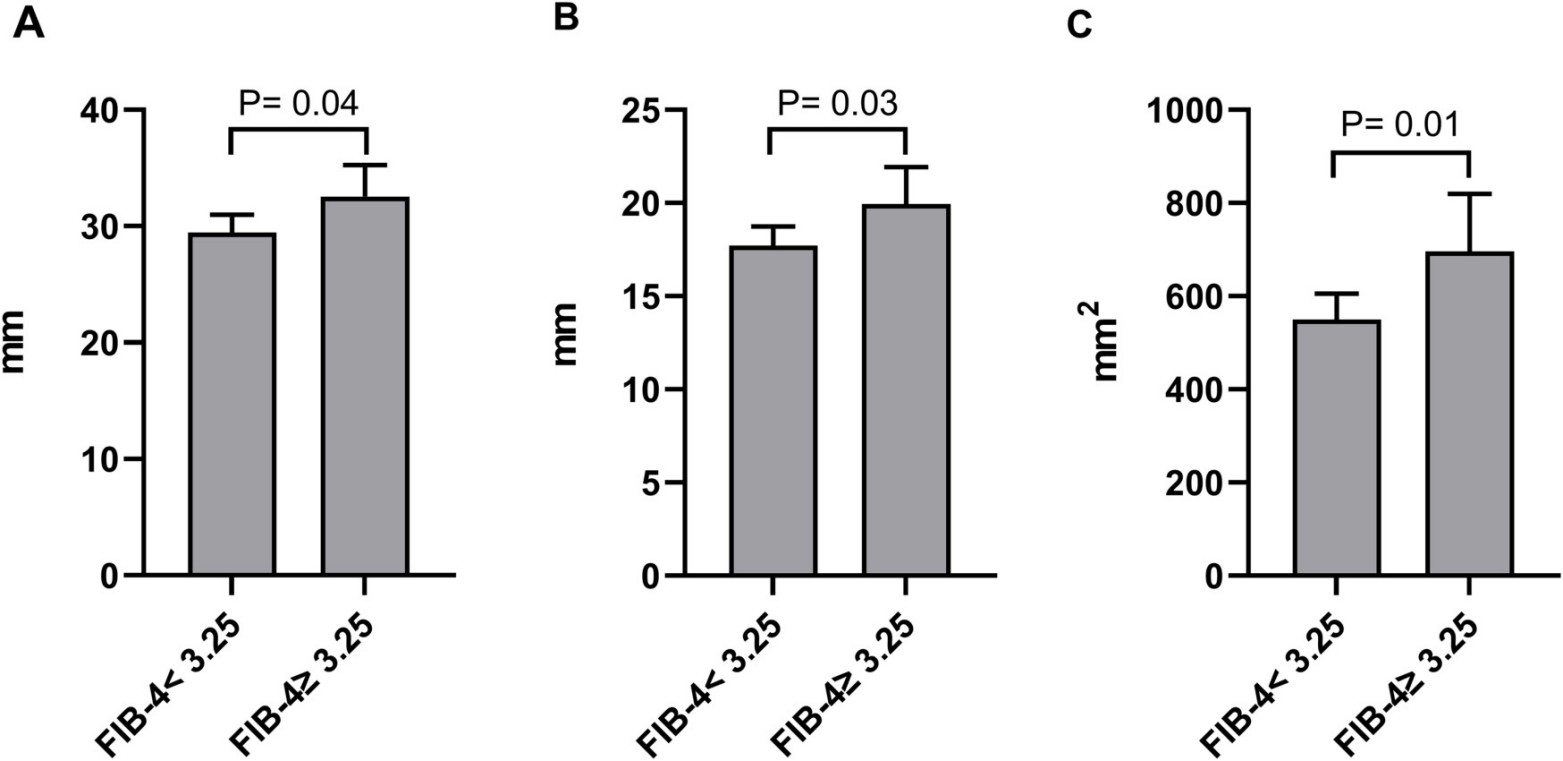
Comparison of the maximal length **(A)**, maximal width **(B)**, and product of length and width **(C)** of LVA between patients grouped by FIB-4 index.

Based on echocardiographic findings, we conducted a comparison of LVA dimensions between patients categorized as FIB-4 < 3.25 and those with FIB-4 ≥ 3.25. As illustrated in Figures 6A–C, patients with FIB-4 ≥ 3.25 demonstrated a notable increase in maximal length, maximal width, and area (calculated as the product of length and width) in contrast to patients with FIB-4 < 3.25 (29.5 ± 8.1 vs. 32.5 ± 8.8 for maximal length, 17.7 ± 5.4 vs. 19.9 ± 6.4 for maximal width, and 549.8 ± 293.3 vs. 696.3 ± 400.3 for the area).

### ROC analysis

**Figure 7:**
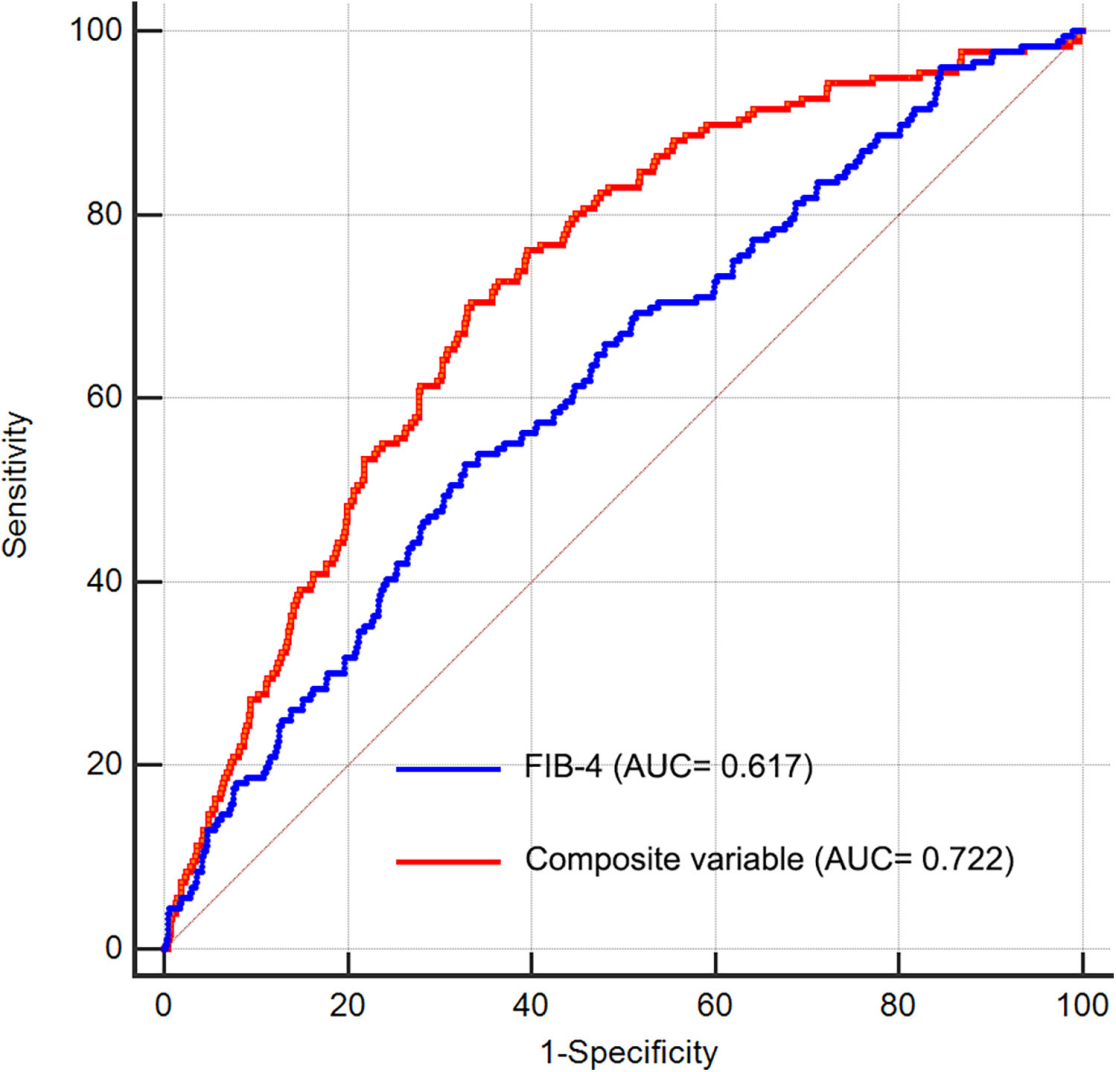
Receiver operating characteristic curve analysis of the FIB-4 index to predict LVA formation. AUC, area under the curve; FIB-4, fibrosis-4.

We performed ROC analysis to evaluate the discriminative capability of the FIB-4 index and composite variable (FIB-4 combined with LVEF, eGFR, and LAD as culprit vessel) in predicting the risk of LVA formation. As shown in Figure 7 and Table 4, the average AUCs for FIB-4 index and the composite variable were 0.617 (95% CI = 0.591–0.642) and 0.722 (95% CI = 0.698–0.746), respectively. The discrepancy between FIB-4 index and the composite variable was statistically significant (*P* < 0.001), indicating that combining FIB-4 index with LVEF, eGFR, and LAD as culprit vessel notably improved the predictive accuracy.

**Table 4:** ROC curve analysis.

Variables	AUC	SE	Sensitivity	Specificity	95% CI
FIB-4	0.617	0.0226	0.54	0.68	0.591–0.642
Composite variable	0.722	0.0195	0.71	0.66	0.698–0.746

ROC, receiver operating characteristic; FIB-4, Fibrosis-4; AUC, area under curve; SE, standard error; CI, confidence interval.

## Discussion

In this study of 1,384 patients hospitalized due to acute STEMI and who underwent primary PCI, we discovered that a greater FIB-4 index correlated with a heightened risk of LVA formation. The statistical significance persisted following a multivariate logistic regression analysis. In our stratified analysis, the influence of other covariates—aside from diabetes—on the association between FIB-4 and LVA risk was minimal. Among patients identified with LVA, individuals with a FIB-4 index of 3.25 or greater displayed significantly larger maximal length, maximal width, and area of LVA when compared to those with a FIB-4 index below 3.25. The combination of FIB-4 index, LVEF, eGFR, and LAD as culprit vessel could substantially improve the ability to distinguish LVA formation.

LVA often occurs as a complication following an acute myocardial infarction, with morbidity rates ranging from 5% to 15% (6). In the context of our research, we found that the incidence of LVA was 12.7%. This finding aligns with previously published data (6, 14). FIB-4, a highly-sensitive biomarker for advanced liver fibrosis, has been linked to various cardiovascular diseases. A study comprising 12,380 patients with non-alcoholic fatty liver disease demonstrated that a FIB-4 score of ≥2.67 correlated with a heightened risk of major adverse cardiac events (MACE) and increased cardiovascular mortality (15). Another study by Nakashima et al. revealed that an elevated FIB-4 index was linked to right ventricular dysfunction, as well as a greater likelihood of subsequent MACE in individuals diagnosed with heart failure with preserved ejection fraction (HFpEF) (12). A prospective, single-center analysis involving 414 patients who suffered from ischemic strokes indicated that the FIB-4 score was independently linked to atrial fibrillation (16). Furthermore, elevated FIB-4 values were connected to higher all-cause mortality risks in patients with acute myocardial infarction (10). Similar to the previous studies, our research indicated that the FIB-4 index was significantly elevated in the LVA group compared to the non-LVA group. Specifically, individuals with FIB-4 ≥ 3.25 had a higher prevalence of LVA than those with FIB-4 < 3.25. Additionally, our analysis using univariate and multivariate logistic regression demonstrated a notably link between the FIB-4 index and the risk of LVA development. RCS analysis also presented a nonlinear association between FIB-4 index and LVA formation after adjusting for possible confounders.

Numerous predictors have been recognized for assessing the risk of LVA development in individuals suffering from AMI. Ran et al. pinpointed the monocyte to HDL cholesterol ratio as a significant predictor of LVA formation in patients experiencing STEMI (2). A case-control study involving 193 patients revealed that abnormalities in the glomerular filtration rate and serum ferritin level served as independent risk factors for LVA after AMI (3). In a retrospectively cohort study comprising 1,823 STEMI patients, factors such as female gender, peak levels of NT-pro BNP, the duration from the onset of pain to balloon intervention, the presence of QS-waves on the initial electrocardiogram, and regional wall motion abnormalities (RWMA) in the left ventricular anterior wall and apex emerged as independent predictors for early LVA (17). Research conducted by Celebi et al. indicated that plasma levels of N-terminal pro B-type natriuretic peptide at the time of admission offered significant predictive insights concerning the onset of LVA following acute STEMI (18). Nevertheless, inconsistencies are present concerning these risk factors due to variations in population data. In this research, we introduced the FIB-4 index as a novel predictor of LVA formation in patients experiencing acute STEMI, with FIB-4 ≥ 3.25 increasing the risk of LVA by 73%. In addition, our study found that LVEF and eGFR also showed significant correlations with LVA formation, aligning with earlier reports in the literature (3, 5). Furthermore, the maximal length, maximal width and area of LVA in patients with FIB-4 ≥ 3.25 were significantly increased compared to those with FIB-4 < 3.25, highlighting the crucial role of the FIB-4 index in predicting the severity of LVA. Combining the FIB-4 index with LVEF, eGFR, and LAD as culprit vessel could substantially improve the predictive accuracy for LVA formation.

It is noteworthy that the odds ratio is higher in patients without diabetes compared to those with diabetes. As illustrated in Figure 5, the association between FIB-4 and LVA does not reach statistical significance among diabetic patients (*P* = 0.122), likely due to the small number of participants with diabetes (*N* = 403). This limitation may also explain the elevated odds ratio found in non-diabetic patients relative to those with diabetes. It is plausible that, with a sufficiently large sample size, the odds ratio for the diabetic population could surpass that of the non-diabetic group.

Our study has certain limitations that should be taken into account. Firstly, it is essential to highlight that the research was performed at a single center and involved a relatively small sample size. This limitation may restrict the applicability of our findings to a broader population and diminish the statistical power of our conclusions. To overcome this concern, it would be beneficial to conduct a multicenter prospective study that includes a larger cohort of participants. Secondly, the methodology for diagnosing LVA in our study was primarily based on ultrasonographic examination. While this technique is widely utilized in both clinical practice and epidemiological investigations due to its accessibility and non-invasive nature, it is crucial to acknowledge that it does not represent the gold standard for the detection of LVA. An alternative diagnostic approach may need to be considered in future studies to establish a more accurate assessment of LVA. Finally, the findings from our study raise questions regarding the causal relationship between an increased FIB-4 index and the formation of LVA. Although our results suggest a correlation, the evidence we gathered does not definitively support a causal link. Further research is necessary to explore this relationship comprehensively and clarify the mechanisms involved in the association between FIB-4 index levels and LVA development.

## Conclusion

This study presents the first evidence that the FIB-4 index is a valuable predictor of LVA formation in patients with acute STEMI who underwent primary PCI. The combination of FIB-4 index, LVEF, eGFR, and LAD as culprit vessel could substantially improve the ability to distinguish LVA formation. These results may offer a novel reference point for the timely recognition of high-risk AMI patients, thereby aiding in the prevention of LVA formation.

## Data Availability

The original contributions presented in the study are included in the article/Supplementary Material, further inquiries can be directed to the corresponding authors.
